# Translation, cross-cultural adaptation and validation of the Diabetes Empowerment Scale – Short Form

**DOI:** 10.1590/S1518-8787.2017051006336

**Published:** 2017-02-21

**Authors:** Fernanda Figueredo Chaves, Ilka Afonso Reis, Adriana Silvina Pagano, Heloísa de Carvalho Torres

**Affiliations:** I Programa de Pós-Graduação em Enfermagem. Escola de Enfermagem. Universidade Federal de Minas Gerais. Belo Horizonte, MG, Brasil; II Departamento de Estatística. Instituto de Ciências Exatas. Universidade Federal de Minas Gerais. Belo Horizonte, MG, Brasil; III Laboratório Experimental de Tradução. Faculdade de Letras. Universidade Federal de Minas Gerais. Belo Horizonte, MG, Brasil; IV Departamento de Enfermagem Aplicada. Escola de Enfermagem. Universidade Federal de Minas Gerais. Belo Horizonte, MG, Brasil

**Keywords:** Diabetes Mellitus, Cost of Illness, Surveys and Questionnaires, Translations, Reproducibility of Results, Validation Studies

## Abstract

**OBJECTIVE:**

To translate, cross-culturally adapt and validate the Diabetes Empowerment Scale – Short Form for assessment of psychosocial self-efficacy in diabetes care within the Brazilian cultural context.

**METHODS:**

Assessment of the instrument’s conceptual equivalence, as well as its translation and cross-cultural adaptation were performed following international standards. The Expert Committee’s assessment of the translated version was conducted through a web questionnaire developed and applied via the web tool e-Surv. The cross-culturally adapted version was used for the pre-test, which was carried out via phone call in a group of eleven health care service users diagnosed with type 2 diabetes mellitus. The pre-test results were examined by a group of experts, composed by health care consultants, applied linguists and statisticians, aiming at an adequate version of the instrument, which was subsequently used for test and retest in a sample of 100 users diagnosed with type 2 diabetes mellitus via phone call, their answers being recorded by the web tool e-Surv. Internal consistency and reproducibility of analysis were carried out within the statistical programming environment R.

**RESULTS:**

Face and content validity were attained and the Brazilian Portuguese version, entitled *Escala de Autoeficácia em Diabetes – Versão Curta*, was established. The scale had acceptable internal consistency with Cronbach’s alpha of 0.634 (95%CI 0.494– 0.737), while the correlation of the total score in the two periods was considered moderate (0.47). The intraclass correlation coefficient was 0.50.

**CONCLUSIONS:**

The translated and cross-culturally adapted version of the instrument to spoken Brazilian Portuguese was considered valid and reliable to be used for assessment within the Brazilian population diagnosed with type 2 diabetes mellitus. The use of a web tool (e-Surv) for recording the Expert Committee responses as well as the responses in the validation tests proved to be a reliable, safe and innovative method.

## RESUMO

**OBJETIVO:**

Traduzir, adaptar culturalmente e validar o *Diabetes Empowerment Scale* – *Short Form* para aplicação no contexto cultural brasileiro.

**MÉTODOS:**

A análise do instrumento, para avaliar a equivalência conceitual e de itens, bem como sua tradução e adaptação cultural, foram realizadas de acordo com a metodologia padrão. A etapa de avaliação pelo comitê de juízes foi conduzida por meio de questionário eletrônico, desenvolvido e aplicado pela ferramenta *web e-Surv*. A versão adaptada foi utilizada durante o pré-teste, aplicado via ligação telefônica, a um grupo de 11 usuários com diabetes melito tipo 2. Os resultados do pré-teste foram examinados por profissionais da área da saúde, linguística aplicada e estatística, para obtenção de uma versão adequada do instrumento. Em seguida, foi aplicada no teste e reteste em amostra de 100 usuários com diabetes, por ligação telefônica, com registro das respostas dos usuários por meio da ferramenta *e-Surv*. As análises de consistência interna e reprodutibilidade foram realizadas no ambiente de programação estatística R.

**RESULTADOS:**

Foi possível obter validade de face e de conteúdo do instrumento, que resultou na versão em português, intitulada Escala de Autoeficácia em Diabetes – Versão Curta. A escala apresentou consistência interna aceitável, com alfa de Cronbach igual a 0,634 (IC95% 0,494–0,737), enquanto a concordância do escore total nos dois momentos foi considerada moderada (0,47). O coeficiente de correlação intraclasse teve o valor de 0,50.

**CONCLUSÕES:**

O processo de tradução e de adaptação para a língua portuguesa falada no Brasil gerou uma versão do instrumento considerada válida e confiável para a população brasileira. A utilização do *e-Surv* para o registro dos dados coletados do comitê de juízes, assim como das respostas dos testes de validação, mostrou-se uma metodologia confiável, segura e inovadora.

## INTRODUCTION

Diabetes mellitus is one of the most common chronic conditions in the world. Brazil has the highest prevalence in South America with about 11.9 million people. Educational practices in relation to diabetes are intended to promote health service users taking responsibility for their own care. This can reduce risk of complications and increase their quality of life^1^. These educational practices cover behavioral, psychosocial and clinical aspects of the heath service users with diabetes^2^ ^,^ ^a^.

Experts from the University of Michigan, USA, prepared, adapted and validated the Diabetes Empowerment Scale (DES), originally made up of 37 questions, into a short version named Diabetes Empowerment Scale – Short Form (DES-SF). The abridged instrument is made up of eight questions addressing the evaluation of psychosocial empowerment and covers the following domains: need for behavior change, development of a care plan, overcoming barriers, soliciting support, caring for oneself, managing emotions, personal motivations, and making decisions about diabetes care^1^ ^,^ ^3^ ^,^ ^4^.

The DES-SF stands out among the instruments that evaluate empowerment in diabetes^10^ ^,^ ^17^, being effective, brief, practical and quickly administered. It can be implemented through telephone call to a large number of users, before and after an educational intervention^2^ ^,^ ^3^.

A group of researchers at the School of Nursing, together with the Laboratory for Experimentation in Translation of the Faculdade de Letras and the Department of Statistics at Universidade Federal de Minas Gerais (UFMG), has been carrying out studies on the translation, adaptation and validation of health instruments within the scope of the project *Empodera – Inovação metodológica nas práticas educativas orientadas à autonomia no cuidado em saúde* (Empodera *–* Methodological innovation in educational practices oriented to autonomy in health care). The group detected the need to use DES-SF in educational practices aimed at assessing users’ psychosocial aspects and to provide health professionals with an instrument that evaluates empowerment in diabetes^b^.

This paper reports a study carried out in order to translate, cross-culturally adapt, and validate the DES-SF for application in the Brazilian cultural context.

## METHODS

Authorization was first requested and obtained from the main author^1^ ^,^ ^8^ of the DES-SF so as to translate, cross-culturally adapt and validate the instrument for Brazilian health service users.

A first analysis of the original instrument explored concepts related to diabetes to verify if the instrument dimensions were relevant to the Brazilian cultural context. A comprehensive bibliographic review as well as interdisciplinary meetings of health and applied linguistics professionals and members of the Center for Management, Education and Health Assessment (NUGEAS) of the Nursing School of the UFMG were carried out for discussion and instrument evaluation.

Once the feasibility and pertinence of using DES-SF in Brazil was ascertained, two Brazilian translators, with a first degree in Translation and Linguistic Studies, were requested to produce a forward translation. The two Brazilian Portuguese versions thereby obtained were compared and a synthesis version generated, which was submitted to back-translation. A professor of Translation and Linguistic Studies, specialized in translation and cross-cultural adaptation of medical instruments, analyzed the originals and back-translations and generated a final version carefully checked to ensure semantic equivalence.

A consolidated final version was obtained, which was submitted for examination to an interdisciplinary committee of experts, with 19 applied linguistics professionals and 19 health professionals. A questionnaire to assess the translated version was used through the web tool e-Surv^5^. Each item in English was followed by its translation and a text box was provided for the experts to paraphrase in their own words what the question meant. After that, experts were asked: a) “Do you think the Portuguese text is in line with the English text?” and b) “Do you think the proposed translation is clear and easy for the respondent to understand it?” The response options were: “Yes”, “no” and “partially”. If “no” and “partially” were the chosen answers, the respondent was requested to point out inadequacies and provide suggestions to improve the translated item. Face and content validity of the instrument were analyzed.

For the pre-test, the version obtained after the committee’s assessment was used and the questionnaire was applied via telephone call to a group of 11 users with type 2 diabetes mellitus. Users were male and female, ranging from 30 to 75 year old and able to listen and verbally respond to the questions in the instrument. Patients with diabetes chronic complications such as chronic dialysis renal insufficiency; blindness; limb amputation and severe psychiatric disorders were not excluded from the sample.

A questionnaire on the web tool e-Surv was used to record users’ comprehension of the translated questionnaire items, with indication of the following options to rate comprehension: 1) The user understood the statement easily; 2) The user had difficulty in understanding the statement; 3) The user asked for the statement to be repeated more than once; and 4) The user was unresponsive to the statement. After three applications of the translated questionnaire with different groups of users, results were discussed by a group of specialists, made up of applied linguistics, health and statistics professionals, whose research focused on cultural adaptation of instruments and diabetes. Adjustments in the wording of some of the items were made in order to facilitate users’ comprehension. After four meetings, the final version of the instrument was achieved (Table 1).

**Table 1:** Original version and final version of the instrument, translated and cross-culturally adapted. Brazil, 2015.

Original version	Final version
*Diabetes Empowerment Scale-Short Form (DES-SF)*	Escala de Autoeficácia em Diabetes Versão Curta (EAD-VC)
*The 8 items below constitute the DES-SF. The scale is scored by averaging the scores of all completed items (Strongly Disagree =1, Strongly Agree = 5)*	Eu (profissional de saúde) vou falar algumas questões sobre como o(a) senhor(a) está cuidando do diabetes. E ai o senhor/a senhora me fala se:
*Check the box that gives the best answer for you*	O senhor / A senhora:
*Strongly Disagree*	Não está de acordo de jeito nenhum
*Somewhat Disagree*	Não está de acordo
*Neutral*	Não tem opinião
*Somewhat Agree*	Está de acordo
*Strongly Agree*	Está muito de acordo
*In general, I believe that I:*	Em geral, eu acredito que:
*1. ...know what part(s) of taking care of my diabetes that I am dissatisfied with.*	1. O(A) senhor(a) sabe que coisas tem de fazer para cuidar da sua saúde, mas não gosta de fazer.
*2. …am able to turn my diabetes goals into a workable plan.*	2. O(A) senhor(a) pode programar o seu dia a dia com coisas que vão ajudar o(a) senhor(a) a cuidar da sua saúde.
*3. ...can try out different ways of overcoming barriers to my diabetes goals.*	3. O(A) senhor(a) pode tentar coisas diferentes para afastar as dificuldades e fazer o que disse que ia fazer para controlar o diabetes.
*4. ...can find ways to feel better about having diabetes.*	4. O(A) senhor(a) acredita que tem como achar coisas diferentes para fazer e sentir bem.
*5. ...know the positive ways I cope with diabetes-related stress.*	5. O(A) senhor(a) pode viver bem e dar um jeito de ir levando esse estresse todo do diabetes.
*6. ...can ask for support for having and caring for my diabetes when I need it.*	6. Quando precisar tem como o(a) senhor(a) pedir ajuda para cuidar do diabetes.
*7. ...know what helps me stay motivated to care for my diabetes.*	7. O(A) senhor(a) sabe o que faz o(a) senhor(a) ficar mais motivado para cuidar do diabetes.
*8. ...know enough about myself as a person to make diabetes care choices that are right for me.*	8. O(A) senhor(a) sabe bem como é que o(a) senhor(a) é, não sabe? Então, dá para o(a) senhor(a) escolher direitinho o que vai dar certo para o(a) senhor(a) cuidar da sua saúde.

This version was applied to 100 users with type 2 diabetes mellitus via telephone call. Simple random sampling process was used and the G-Power software to calculate sample size. A 5% significance level, a 80.0% test power, equal standard deviations in the test and retest scores, and a 0.30 correlation coefficient (minimum value detected in the consistency assessment) were considered; the minimum sample size was 82 users. Considering the friction rate of no more than 20.0%, 100 users made up the initial sample^9^.

The instrument was applied twice (test and retest) with a 15-day interval as recommended in the literature for this validation step^8^ ^,^ ^23^. The same interviewer conducted the first and second interviews at the same scheduled times.

The interviewer recorded the answers in a questionnaire using the tool web e-Surv^c^, with a questionnaire made up by the following sections: 1) Instructions for the interviewer; 2) Sociodemographic and clinical data of the user; 3) translated and cross-culturally adapted instrument; and 4) Interviewer’s remarks.

In section 1, the interviewer welcomed the user, explained the study, especially its aims, obtained their consent to participate, provided answers to user inquiries, and requested permission to record the call. In section 2, the interviewer collected sociodemographic data and clinical information, such as when diabetes had been diagnosed, type of treatment and health problems. In section 3, the interviewer read each question from the adapted version of the instrument. The Likert scale used was “I totally agree” – 5 points; “I agree” – 4 points; “I have no opinion” – 3 points; “I disagree” – 2 points; and “I totally disagree” – 1 point. The final score was estimated by the mean of the scores in the eight questions; high score being from 3.8 to 5.0, moderate from 2.4 to 3.7 and low from 1 to 2.3^1^.

Sample data was analyzed according to sociodemographic and clinical variables. The Cronbach’s alpha coefficient (internal consistency) and the Spearman’s correlation coefficient (correlation between the responses to the instrument items at test and retest times) were used as a measure of agreement between two measurements for the reliability assessment. The latter was chosen due to its lower sensitivity to discrepant values, compared to its parametric version^6^. The intraclass correlation coefficient (ICC) was also used as a measure of agreement between the total score in two instrument applications, while the Wilcoxon test verified statistical difference between the median score of the first and second applications of the instrument. The significance level for statistical inference was 5.0%^15^.

The data were stored with code identifier in a data spreadsheet and imported for analysis within the statistical programming environment R^d^.

The Ethics Committee on Research Involving Human of the Health Secretariat of Belo Horizonte and the Ethics Committee on Human Research of the UFMG (Opinion 1,018,006/2015) granted approval to this study. Before recording the telephone calls, the users’ consent to participate in the study and to answer the questionnaire questions was recorded.

## RESULTS

The analysis of conceptual and item equivalence between the original and the translated and the cross-culturally adapted versions contributed to the consolidation of a consensual version of the scale in Brazilian Portuguese, called *Escala de Autoeficácia em Diabetes – Versão Curta* (EAD-VC).

The expert committee assessment found question 6 to have a high frequency of answers indicating the need to adapt the proposed translation from English into Portuguese, with 31.6% in the group of linguistic studies specialists and 57.9% in the group of healthcare professionals. In the other questions, “partially” and “no” answers in the group of healthcare professionals were more frequent. Respondents suggestions were extensively discussed until obtaining the final version.

The final version was used for the pre-test in a sample of 11 health service users. 60.0% of the users found it difficult to understand three questions (2, 6 and 7), the interviewer having been requested to repeat the question and explain it. Therefore, these questions were reworded into versions with more easily understood items, such as using “motivated” rather than “animated”, “care” rather than “treat”, “program” instead of “plan.” The first person pronoun (“I”) was changed into the third person and a politeness marker used “sir/madam”, so as to facilitate the interviewer’s spoken interaction with the user and the application via telephone call.

To analyze the instrument reliability, a 100-user sample of Basic Health Unit users with diabetes mellitus type 2 was used. Among the interviewees, 38.0% had incomplete basic education and 66.0% were inactive (retired or homemaker) (Table 2). The mean age was 61.3 years (SD = 8.7 years), while the mean length of diabetes condition was 10 years (SD = 7.6 years). The mean self-efficacy in the test was 4.4 (SD = 0.5) and, in retest, 4.4 (SD = 0.4) (Table 3).

**Table 2:** Characterization of the sample studied for validation of the Diabetes Empowerment Scale – Short Form (DES-SF). Brazil, 2015.

Characteristic		Frequency	%
Sex	Male	39	39.0
	Female	61	61.0
Schooling	Illiterate	8	8.0
	Incomplete elementary school	38	38.0
	Complete elementary school	34	34.0
	Incomplete high school	2	2.0
	Complete high school	14	14.0
	Higher education	4	4.0
Occupation	Active	34	34.0
	Inactive	66	66.0
Marital status	With no partner	36	36.0
	With a partner	64	64.0
Treatment	No use of Insulin and Oral agents	1	1.0
	Insulin	16	16.0
	Oral agents	17	17.0
	Insulin and Oral agents	36	36.0

Total		100	100

**Table 3:** Absolute frequency distribution of scores for each instrument question at test and retest times. Brazil, 2015.

Variable	Assessment	Score				

		1	2	3	4	5
Question 1	Test	14	13	2	47	24
	Retest	21	3	0	38	38
Question 2	Test	1	6	0	30	63
	Retest	2	4	2	20	72
Question 3	Test	0	0	0	51	45
	Retest	2	1	0	27	70
Question 4	Test	1	8	0	35	56
	Retest	1	2	0	22	75
Question 5	Test	3	1	0	45	51
	Retest	6	2	0	24	68
Question 6	Test	8	3	1	19	69
	Retest	4	5	0	18	73
Question 7	Test	4	2	3	27	64
	Retest	6	1	2	9	82
Question 8	Test	1	1	0	25	73
	Retest	0	0	0	16	84

The Cronbach’s alpha assessed the internal consistency considering the first application data, before the users had any contact with the instrument. The *Escala de Autoeficácia em Diabetes – Versão Curta* (EAD-VC) presented a Cronbach’s alpha = 0.634 (95%CI 0.494–0.737) and its internal consistency was acceptable.

Cronbach’s alpha took one question at a time (Table 4), to evaluate the influence of each item on the internal consistency of the instrument. Comparing only the values of the alpha point estimates, just the removal of item 1 increased the value of the alpha point estimate. Item 1 had the highest rates of discordance in the test and in the retest, with more divergent responses than the answers to the other items. With the removal of item 1, the total score of the scale became more homogeneous, increasing the alpha index.

**Table 4:** Correlation between test and retest responses (item by item and total score) and Cronbach’s alpha *coefficient (*α) for Diabetes Empowerment Scale – Short Form. Brazil, 2015.

Questions	Spearman’s Correlation *Coefficient* – test and retest	Cronbach’s *alpha* if the item is removed	95%CI
1	0.24	0.709	0.561–0.803
2	0.28	0.602	0.449–0.709
3	0.10	0.592	0.424–0.710
4	0.51	0.585	0.419–0.692
5	0.18	0.595	0.453–0.700
6	0.51	0.542	0.377–0.662
7	0.40	0.577	0.400–0.687
8	0.06	0.606	0.457–0.711
Total score	0.47	0.634	0.494–0.737

The instrument reliability used the correlation between the responses to each item of the test and the retest with the Spearman’s correlation coefficient (Table 4). Despite the low value of this coefficient in some questions (3, 5 and 8), the total score concordance was considered moderate (0.47). For questions 3 and 8, responses in the “agree” and “strongly agree” categories in the test (question 3) or in the retest (question 8) were higher (Table 3). Furthermore, examining the cross-over of answers to questions 3 and 8 in the test and retest (not shown in the text) showed that most users who said they were “in agreement” or “agree a lot” at the first moment also chose one of these two responses in the second moment (97.0% and 100%, respectively). However, this high concentration of answers in two categories at one of the moments caused many draws at the stations used to calculate the Spearman’s coefficient, leading to very low values for the coefficient in these questions, since it is sensitive to draws.

The analysis of the intraclass correlation coefficient supported the test-retest reliability of the instrument after two weeks, with moderate agreement (ICC = 0.50; 95%CI 0.495–0.504).

The median score at the time of retest was higher than the median test score (difference between the medians equal to 1 point; Wilcoxon test, p = 0.013). However, by reducing the responses to three options (“I disagree or agree a lot”, “I have no opinion” and “I agree or strongly agree”), the difference between the median scores was statistically insignificant (difference between the sample medians equal to zero; Wilcoxon test, p = 0.757).

Both applications showed a difference in response time to the instrument in the test-retest. All users took five to ten minutes to complete the test and less than five minutes to complete the retest. Sociodemographic and clinical data were collected in the second section of the e-Surv questionnaire.

## DISCUSSION

The evaluation of conceptual and item equivalence led to the translated and cross-culturally adapted version of the instrument that is viable and practical for educational actions geared towards diabetes care in Brazil. Face and content were found adequate. The expert committee and the test and pre-test users found the understanding of the items satisfactory. The test and pre-test users had various levels of schooling, a criterion adopted to determine which language was appropriate for all levels, following the recommendations in the literature^12^ ^,^ ^13^ ^,^ ^19^. The instrument was found to be suitable as spoken text and to facilitate interaction and user comprehension as well as adequate to the Brazilian cultural context^7^ ^,^ ^18^.

Internal consistency analysis provided Cronbach’s alpha value of 0.634 (95%CI 0.494–0.737). This result is acceptable in the literature^23^. The studies^13^ ^,^ ^14^ ^,^ ^16^ ^,^ ^20^, which validated the original version of the instrument with 37 questions, showed Cronbach’s alpha values above 0.70. Studies describing the validation of translated and adapted DES-SF versions are not available in the literature in order to compare with our results. Moreover, Tavakol and Dennick^23^ (2011) state that the Cronbach’s alpha value is affected by the size of the instrument, a short instrument tending to have a low alpha value.

The retest median score can be equal to the test median score, indicating no statistically significant changes in the users’ empowerment between the test and the retest.

There are different instruments in the literature that assess empowerment in diabetes^10^ ^,^ ^17^ ^,^ ^22^. However, the *Escala de Autoeficácia em Diabetes – Versão Curta* (EAD-VC) stands out for its ease and feasibility of application. Most significantly, its application via telephone call is viable; the instrument can be also used as a process and result indicator of educational practices in diabetes, which allows for longitudinal care^24^ ^,^ ^25^.

The validation process of this study provided three main findings. The first, made during the application of the instrument, concerns the need to problematize the relevance and adequacy of a *Likert scale* with five options. This is because users tend to agree or disagree with a given question without, however, being able to quantify the intensity of such agreement or disagreement. This confirms observations in previous studies and it suggests the need to adapt the scale to three options^10^ ^-^ ^12^ ^,^ ^21^. However, the number of categories of the scale in this study was kept to five so that the results of the DES-SF application were comparable to studies in other countries^2^ ^,^ ^3^.

We observed the second finding in the retest application, in which some users answered differently some of the questions. A study^18^ that focused on the respondents’ behavior from communities of Latin American descendants in the United States pointed to randomness in the participants’ responses with low schooling and advanced age (> 60 years). Similar characteristics of the users interviewed in this study may explain the moderate ICC outcomes (0.50) and Spearman (0.47).

The third finding concerns the use of a questionnaire implemented through a web tool such as e-Surv, which proved to be a reliable, safe and innovative methodology. It allowed for consolidating responses by healthcare professionals, applied linguistics specialists and statistics specialists during the cross-cultural adaptation of the instrument, during the consultation to the expert committee; it also offered the possibility of experts participation without the need to relocate or leave their offices. This also favored the feeling of anonymity and allowed the participants to express certain opinions that they would not dare to if the whole procedure had been carried out in face-to-face interaction^5^. The tool also facilitated data collection in the validation tests, since the spreadsheet with the interview information is generated automatically, only requiring few adjustments to import and analysis within the statistical programming environment R^17^.

This study contributed to provide a comprehensible and feasible instrument to be applied via telephone call to a large number of users for the evaluation of psychosocial empowerment. This is meant to improve diabetes care in Brazil^16^.

The need to carry out further studies to obtain further evidence of EAD-VC validity and reliability, using other representative samples, including healthcare users from different regions in Brazil, is a limitation of this study. This limitation will be solved in a continuous process of instrument evaluation; this process must be sensitive to the evolution of the instrument construct.

## Footnotes

a International Diabetes Federation. Diabetes atlas. Brussels; 2014 [cited 2015 Mar 19]. Available from: http://www.idf.org/diabetesatlas

b Torres HC, Reis IA, Pagano AS. Empoderamento do pesquisador nas ciências da saúde. Belo Horizonte (MG): FALE/UFMG; 2015.

c e-Surv. Reino Unido; 2001 [cited 2015 Jan 6]. Available from: https://eSurv.org?s=LIEJOO_dcfefe5e

d R Development Core Team. R Foundation for Statistical Computing. Viena; 2014 [cited 2015 Jan 20]. Available from: http://www.R-project.org/
